# Death domain-associated protein (Daxx) impairs colon cancer chemotherapy by inhibiting the cGAS-STING pathway

**DOI:** 10.32604/or.2024.054930

**Published:** 2025-04-18

**Authors:** XI ZHU, KAI HUANG, XIAOMING KAO, ZHAOHUI TANG, WENJIE GUO, TIANCONG WU, QIURONG LI

**Affiliations:** 1 Research Institute of General Surgery, Jinling Hospital, Nanjing Medical University, Nanjing, 210002, China; 2 Research Institute of General Surgery, Nanjing Jinling Hospital, Affiliated Hospital of Medical School, Nanjing University, Nanjing, 210002, China; 3 State Key Laboratory of Pharmaceutical Biotechnology, School of Life Science, Nanjing University, Nanjing, 210093, China; 4 Department of Radiation Oncology, Nanjing Jinling Hospital, Affiliated Hospital of Medical School, Nanjing University, Nanjing, 210002, China

**Keywords:** Death domain-associated protein (Daxx), Stimulator of the interferon gene (STING), Colorectal cancer (CRC), Chemotherapy

## Abstract

**Background:**

Colorectal cancer (CRC) holds the third position in global cancer prevalence mortality. Although chemotherapy is a conventional treatment, recent investigations have shed light on the therapeutic potential of the cGAS cyclic GMP-AMP synthase (cGAS)-stimulator of interferon genes (STING) pathway in CRC management. Despite the primary role of the death domain-associated protein (Daxx) in cellular apoptosis, its influence on the regulation of cGAS-STING activation remains elusive.

**Methods:**

The Daxx degradation and speck formation were conducted using immunofluorescence and Western blotting. The Daxx knock-down and over-expression in CRC cells were performed to detect *in vivo* and *in vitro* migration, proliferation, cGAS-STING activation, and immune responses.

**Results:**

Our study reveals that treatment with irinotecan (CPT-11) and oxaliplatin (OXA) significantly accelerated the Daxx degradation and diminished the formation of Daxx specks within the nucleus of CRC cells. Genetic elimination of Daxx enhanced the irinotecan and oxaliplatin-induced suppression of proliferation and migration in CRC cells, and overexpression of Daxx resulted in similar results. Mechanistically, Daxx overexpression reduced DNA damage repair by restraining homologous recombination (HR) over non-homologous end-joining (NHEJ), which suppressed TBK1 and IRF3 phosphorylation downstream of the cGAS-STING signal. In a murine model of CT-26 tumors, Daxx knockdown amplified the OXA-mediated tumor growth inhibition by promoting STING activation and immune responses.

**Conclusions:**

Our findings show that the degradation of nuclear Daxx potentiates the cGAS-STING pathway, thereby bolstering the efficacy of chemotherapy.

## Introduction

Globally, colorectal cancer (CRC) holds the position as the third leading cause of cancer-related deaths. Risk factors include alcohol consumption, a diet high in processed or red meats, and a low intake of calcium, fiber, and milk [1–3]. Over 1.2 million patients with CRC are diagnosed per year, with more than 600,000 dying [4,5]. Treatment options encompass local surgical excision, targeted therapy, immunotherapy, chemotherapy, and radiotherapy [6]. Systemic chemotherapy is an essential method in metastatic and adjuvant treatment settings. Chemotherapeutic agents, such as oxaliplatin (OXA), irinotecan, and 5-fluorouracil, are indispensable for treating CRC, often in combination with immunotherapy or targeted drugs [7].

The death domain-associated protein (Daxx) interacts specifically with the Fas death domain, and its overexpression promotes apoptosis mediated with Fas and triggers the Jun N-terminal kinase activation [8]. In B-cell lymphoma, Daxx was regarded as the negative regulator of TGF-β-mediated apoptosis, and antisense oligonucleotides targeting Daxx inhibited TGF-β-mediated apoptosis in murine hepatocytes by interfering with TβRII [9]. Cells with depletion Daxx confer resistance to UV irradiation and oxidative stress-induced cell death in primary fibroblasts, and downregulation of Daxx leads to impaired activation (JNK) activation [10]. Additionally, Daxx negatively regulates hypoxia-induced cell dissemination and invasion by directly binding to the Slug’s DNA-binding domain. This interaction impedes the recruitment of histone deacetylase 1 (HDAC1) and antagonizes Slug E-box binding [11]. However, some studies have indicated that Daxx plays an essential antiapoptotic factor [12]. For instance, Daxx knock-down significantly sensitizes cell apoptosis induced by Fas and stress, accompanied by the activation of caspase and Jun N-terminal kinase [13]. Daxx disruption enhances apoptosis during murine embryonic development, which suggests that Daxx acts as the antiapoptotic protein in embryos [14]. Finally, under an extrachromosomal telomere repeat (ECTR) mutation induced by 4-hydroxytamoxifen (4-OHT), Daxx mediated activation of the DNA-sensing pathway [15]. Daxx expression level is reduced in liver metastases compared to primary colon cancer tissues, and it directly engages with ZEB1 to inhibit cell movement, migration, and invasion [16]. CD24 promotes adhesion and signifies the spread of cancer, whereas Daxx expression positively correlates with CD4 expression in CRC patients [17]. Daxx interacts with Tcf4 and inhibits its transcriptional function, thereby modifying downstream gene expression of Tcf4 and facilitating G1 arrest in colon cancer cells [18,19].

STING, the stimulator of interferon genes, also called MPYS, TMEM173, ERIS, or MITA, serves as the primary sensor to regulate innate immunity [20–22]. Upon detecting double-stranded DNA (dsDNA), cyclic GMP-AMP synthase (cGAS) recognizes the DNA to synthesize cyclic GMP-AMP (cGAMP), which binds to and triggers STING and recruits TANK-binding kinase 1 (TBK1), activating the phosphorylation of interferon regulatory factor 3 (IRF3) and interferon signaling pathway [23–25]. Studies indicate that cGAS and STING silencing can partially restrict the anti-tumor potential induced by DNA damage agents and programmed cell death 1 ligand 1 (PD-L1) antibody [26]. Furthermore, the dephosphorylation of poly (ADP-ribose) polymerase 1 (PARP1) by SH2 domain-containing protein-tyrosine phosphatase-2 (SHP2) inhibits DNA repair and promotes STING-mediated antitumor immunity response, suggesting that tumor intrinsic STING activation is also important for chemotherapy [27–29].

Recent research on STING has expanded to include its role in tumor and immune responses [30–33]. Studies indicate that STING activity is upregulated in metastatic cells but inhibited by hypermethylation of the STING promoter in metastatic lesions [30]. Additionally, irinotecan (CPT-11)-induced intestinal mucositis depends on the cGAS-STING signal triggered by DNA damage [34]. Although the cGAS-STING signal activation holds promise for bolstering antitumor defenses through interferon signaling, its clinical application poses challenges. Clinical trials involving small molecules aimed at STING activation have uncovered the notable side effects from inflammatory cytokine storms caused by excessive STING activation [22,35]. Therefore, exploring methods to relieve endogenous inhibition of STING rather than solely focusing on its direct activation is necessary.

In our study, our results indicated that knockdown Daxx restricted tumor cell proliferation and migration caused by irinotecan and OXA while increasing Daxx expression resulted in a contrary impact. Notably, Daxx overexpression in tumor cells promoted DNA damage repair primarily through homologous recombination rather than nonhomologous end-joining. Conversely, the genetic ablation of Daxx promoted STING activation. Additionally, Daxx knockdown notably enhanced the inhibitory effects of OXA on tumor growth by promoting STING activation and eliciting immune responses *in vivo*. These findings highlight the significance of nuclear Daxx degradation in triggering the cGAS-STING signal to enhance chemotherapy sensitivity, presenting a promising strategy for treating CRC.

## Materials and Methods

### Cell lines

The human colon cancer cell line (CBP60028, HCT-116, and CBP60011, HT-29), mouse colon cancer cell line (CBP61189, CT-26), and human embryonic kidney cell line (CBP60439, HEK-293T) were purchased from the Shanghai Cell Bank of the Chinese Academy of Sciences (Shanghai, China). All cell lines were identified and there was no mycoplasma infection. These cells were cultivated in McCoy’s 5A (01-075-1ACS, Biological Industries, Kibbutz Beit Haemek, Israel), PRMI 1640 (11875085, Gibco, Grand Island, NY, USA), and DMEM high-glucose (01-056-1A, Biological Industries, Kibbutz Beit Haemek, Israel) supplemented with 10% FBS (C04001-500, Biological Industries, Kibbutz Beit Haemek, Israel) and penicillin-streptomycin buffer (C0222, Beyotime, Shanghai, China) and cultured in a humidified incubator (Thermo Fisher Scientific, Waltham, MA, USA) with 5% CO_2_, 37°C. Those cells were exposed to CPT-11 (T6228, TargetMol, Shanghai, China) and OXA (T0164, TargetMol, Shanghai, China).

### Western blotting

HCT-116 cells were treated with OXA and CPT-11, all cells were lysed in RIPA buffer (P0013C, Beyotime, Shanghai, China) with protease inhibitor PMSF (ST505, Beyotime, Shanghai, China) for 0.5 h on ice. Insoluble fractions were isolated by centrifuge (5425R, Eppendorf, Hamburg, Germany) at 12,000 rpm (4°C) for 10 min, and protein supernatant was resuspended in a loading buffer (pH = 6.8) and denatured by boiling (100°C, 10 min). Samples of protein were separated by SDS-polyacrylamide gel electrophoresis (SDS-PAGE) with different concentrations and transferred to PVDF membranes. Following blocking with 5% non-fat milk in TBS-T buffer at room temperature for 1 h, the membranes with proteins were anti-TBK1 (1:1000, 67211-1-Ig, Proteintech, Wuhan, China), anti-p-TBK1 (1:1000, P00261, Boster, Wuhan, China), anti-IRF3 (1:1000, 11312-1-AP, Proteintech, Wuhan, China), anti-p-IRF3 (1:1000, 29047, Cell Signaling Technology, Danvers, MA, USA), anti-HA (1:1000, M20008S, Abmart, Shanghai, China), anti-β-tubulin (1:2000, M20005S, Abmart, Shanghai, China), anti-GAPDH (1:2000, M20006, Abmart, Shanghai, China), anti-Daxx (1:1000, 4533S, Cell Signaling Technology, Danvers, MA, USA) primary antibodies at 4°C overnight followed by washing 3 times in 1×TBS-T (G0004-500ML, Servicebio, Wuhan, China), and then incubated with secondary mouse antibody (1:2000, SA00001-1, Proteintech, Wuhan, China) and rabbit antibody (1:2000, SA00001-4, Proteintech, Wuhan, China) for 2 h at room temperature. Detection was carried out using the chemiluminescence instrument (ChemiDoc XRS+, BIO-RAD, Hercules, CA, USA). GAPDH and β-tubulin normalized densitometry results. For the quantitative analysis of the bands, the optical density was determined using ImageJ2 software (Version 2.1.5.0; National Institutes of Health, Bethesda, MD, USA).

### Cell transfection

HCT-116 cells and HEK293 cells were transfected with some plasmids (pCBASceI, pDRGFP, pimEJ5GFP, and HA-Daxx) using Hieff Trans™ Liposomal Transfection Reagent (40802ES02, Yeasen Biotechnology, Shanghai, China) in DMEM with 10% FBS for 24 h. The quantity of transfection reagents should be according to the operation instructions.

### Cellular immunofluorescence

HT-29 and HCT-116 cells (Cell density: 50%) were incubated with CPT-11 (30 µM) or OXA (8 µM) in PRMI 1640 or McCoy’s 5A with 1% FBS, 4% FPA fixed those cells for 15 min, and the use of 0.2% Triton 100 in 1×PBS (G4202-100ML, Servicebio, Wuhan, China) for 15 min increased cells permeability, and blocking with 5% BSA (GC305010-5 g, Servicebio, Wuhan, China) in 1×PBS-T (Tween 20, GC204002-100ml, Servicebio, Wuhan, China) for 1 h at room temperature. The primary antibody (Daxx antibody, 4533S, Cell Signaling Technology, Danvers, MA, USA, and HA antibody, M20008S, Abmart, Shanghai, China) was exposed to those cells overnight at 4°C. For secondary antibody of fluorescence, Goat anti-Mouse IgG (H+L) Highly Cross-Adsorbed Secondary Antibody, Alexa Fluor 594 (Thermo Fisher Scientific, Waltham, MA, USA), and Goat anti-Rabbit IgG (H+L) Highly Cross-Adsorbed Secondary Antibody, Alexa Fluor 488 (Thermo Fisher Scientific, Waltham, MA, USA) were added and incubated for 2 h in the absence of light at room temperature. Finally, a TCS SP8 confocal microscope (Leica, Wetzlar, Germany) was used to take the cellular fluorescent photos. For quantitative analysis of the Daxx speck, we randomly select four fields to count the average number of Daxx specks in the nucleus.

### Wound-healing assay

CRC cells (HCT-116 and CT-26) were inoculated into 6-well plates (5 × 10^5^) in McCoy’s 5A or PRMI 1640 with 10% FBS and transfected with HA-Daxx plasmid and lentivirus-shDaxx, those cells were scraped to shape a wound by using 200 μL yellow tips with a sterile pipette and treated with CPT-11 and OXA. Images of wound healing were obtained at different time points (0, 24, and 48 h) using the microscope (Olympus, Tokyo, Japan). For quantification of the wound-healing assay, the ImageJ software was performed to calculate the non-healing area and data analysis using Graph Prism (Version 10.0; La Jolla, CA, USA).

### MTT assay

CRC cells (HCT-116) cells were inoculated into 96-well plates (2 × 10^3^/well), and those cells transfected with Human Daxx cDNA ORF clone, pCMV3-C-HA (HA-Daxx) plasmid, or lentivirus-shDaxx and treated with CPT-11(30 µM) and OXA (8 µM) for 48 h at 37°C, 5% CO_2_. The 12 µL MTT (5 mg/mL, ST316, Beyotime, Shanghai, China) per well was added into the cellular culture medium at 37°C for 4 h, 5% CO_2_. Finally, 100 µL DMSO was added into wells, and using a microplate reader (M200Pro, Infinite, Swess) at 570 nm the OD value per wells. A Human Daxx cDNA ORF clone, pCMV3-C-HA plasmid was purchased from Sino Biological Inc. (HG17618-CY, Beijing, China).

### DNA damage repair detection

For homologous recombination, plasmids encoding pCBASceI (1.5 µg/well), pDRGFP (1 µg/well), and HA-Daxx (2 µg/well) were together transfected into HCT-116 and HEK293T cells (Those cells were inoculated into 6-well plates, 5 × 10^5^/well) using the Hieff Trans™ Liposomal Transfection Reagent (40802ES02, Yeasen Biotechnology, Shanghai, China). The vector group: pCBASceI, pDRGFP, and vector plasmids; The OE-Daxx group: pCBASceI, pDRGFP, and HA-Daxx plasmids.

For nonhomologous end-joining, plasmids encoding pCBASceI (1.5 µg/well), pimEJ5GFP (1 µg/well), and HA-Daxx (2 µg/well) were together transfected into HCT-116 and HEK293T cells using the same transfection reagent. The vector group: pCBASceI, pimEJ5GFP, and vector plasmids; The OE-Daxx group: pCBASceI, pimEJ5GFP, and HA-Daxx plasmids. Finally, all cells were digested and resuspended in 1×PBS, and the cellular percentage of GFP-positive was detected using a flow cytometer (Thermo Fisher, Waltham, MA, USA). Data analysis through FlowJo software (Version 10.0; TreeStar, Ashland, OR, USA).

### In vivo allograft mouse model

All female BALB/c mice (16–18 g) were purchased from Charles River (Beijing, China), and all mice live in a pathogen-free barrier facility. All mice stayed with highly free access to food and water in cages at a comfortable temperature of 21 ± 2°C and kept on a light/dark cycle for half a day. Animal welfare and experimental procedures with mice were performed according to the Guide for the Care and Use of Laboratory Animals (Ministry of Science and Technology of China, 2006) and the operating standards of Nanjing Jinling Hospital, Affiliated Hospital of Medical School, Nanjing University. All efforts were used to decrease animals’ suffering and reduce animals’ numbers in our research.

The lentivirus-shDaxx or shctrl was used to infect CT-26 cells, the 6–8 weeks BALB/c mice were subcutaneously implanted with 1 × 10^6^ CT-26 cells (1 × 10^7^/mL in 1×PBS) on day 0. When the mouse tumor grows to 100 mm^3^ measured with a vernier caliper, the mice receive OXA treatment by intraperitoneal administration for 12 days (Twice, day 1 and day 7, respectively). The control mice were injected with saline. Finally, the method of euthanizing mice by cervical dislocation was used, and the tumor tissues were isolated from mice and photographed and weighed on day 12. The groups are as follows: (i) Control; (ii) OXA 5 mg/kg, intraperitoneal administration (Twice, day 1 and day 7, respectively); (iii) ShDaxx; (iv) ShDaxx + OXA 5 mg/kg, intraperitoneal administration (Twice, day 1 and day 7, respectively).

### Hematoxylin-eosin (H&E) staining

CRC tissues from mice were fixed overnight in 4% formalin and were embedded in paraffin, and all samples were cut into tissue sections for 5 µm by microtome (RM2125RTS, Leica, Wetzlar, Germany). Tumor sections were dewaxed by dimethylbenzene and ethanol and were prepared for hematoxylin-eosin (H&E) staining. Subsequently, hematoxylin staining (10 min), tissue differentiation using ammonia, and eosin staining (5 min) were performed at room temperature. Images of H&E staining were captured using a microscope (BX51, Olympus, Tokyo, Japan).

### Statistical analysis

All data are expressed as mean ± SD in three independent experiments, and each experiment included triplicate sets. Statistical data analysis was conducted using a *t*-test and Dunnett’s test between the control and the experimental groups. The significance was set at a *p*-value of 0.05.

## Results

### Chemotherapy drugs promoted the degradation of Daxx

**Figure 1:**
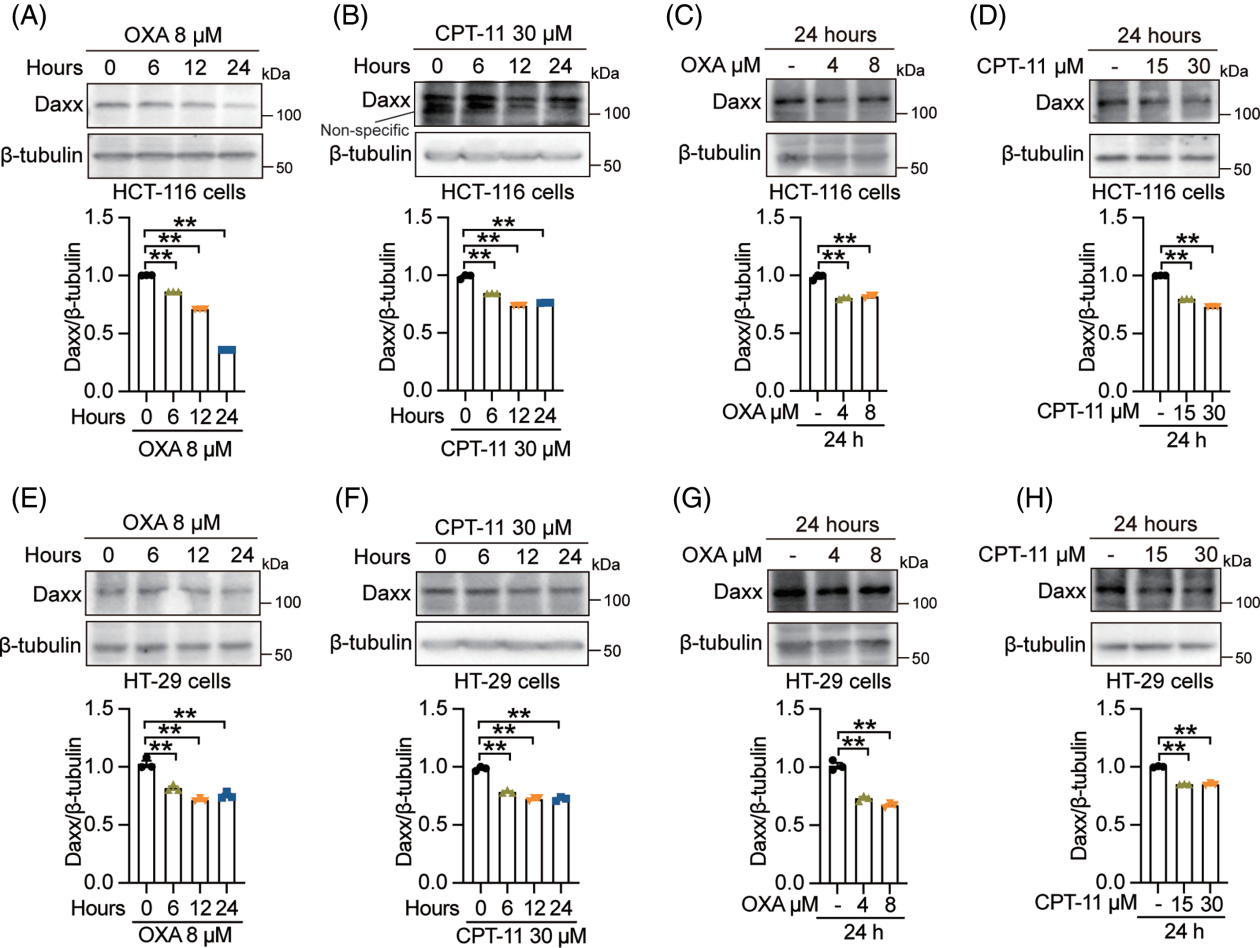
Chemotherapy promoted the degradation of Daxx. (A and C) HCT-116 cells were treated with OXA (8 µM) for 0, 6, 12 and 24 h or OXA (0, 4 and 8 µM) for 24 h. (B and D) HCT-116 cells were exposed to CPT-11 (30 µM) for 0, 6, 12, and 24 h or CPT-11 (0, 15, and 30 µM) for 24h. (E and G) HT-29 cells were incubated with OXA (8 µM) for 0, 6, 12 and 24 h or OXA (0, 4 and 8 µM) for 24 h. (F and H) HT-29 cells were exposed to CPT-11 (30 µM) for 0, 6, 12, and 24 h or CPT-11 (0, 15, and 30 µM) for 24 h. The expression level of Daxx was detected by Western blotting. The circles, triangles, and squares represent different groups, and one group has only one shape. Values were shown as the means ± SEM in A–H. **p < 0.01 *vs*. as indicated.

CPT-11 and OXA are chemotherapeutic drugs approved to treat patients with CRC [36,37]. Our findings indicated that OXA and CPT-11 enhance the Daxx degradation in a time-dependent manner, with low Daxx expression observed at 24 h in HCT-116 cells (Fig. 1A,B). For varying concentrations, OXA and CPT-11 enhanced Daxx degradation in HCT-116 cells (Fig. 1C,D). Similarly, in HT-29 cells, OXA and CPT-11 promote Daxx degradation in a time-dependent manner, with low Daxx expression levels at 24 h (Fig. 1E,F). CTP-11 and OXA also promoted Daxx degradation in different doses in HT-29 cells (Fig. 1G,H). These results suggested that OXA and CPT-11 promoted Daxx degradation in human CRC cells.

### Chemotherapy drugs inhibited the speck formation of Daxx in the nucleus

**Figure 2:**
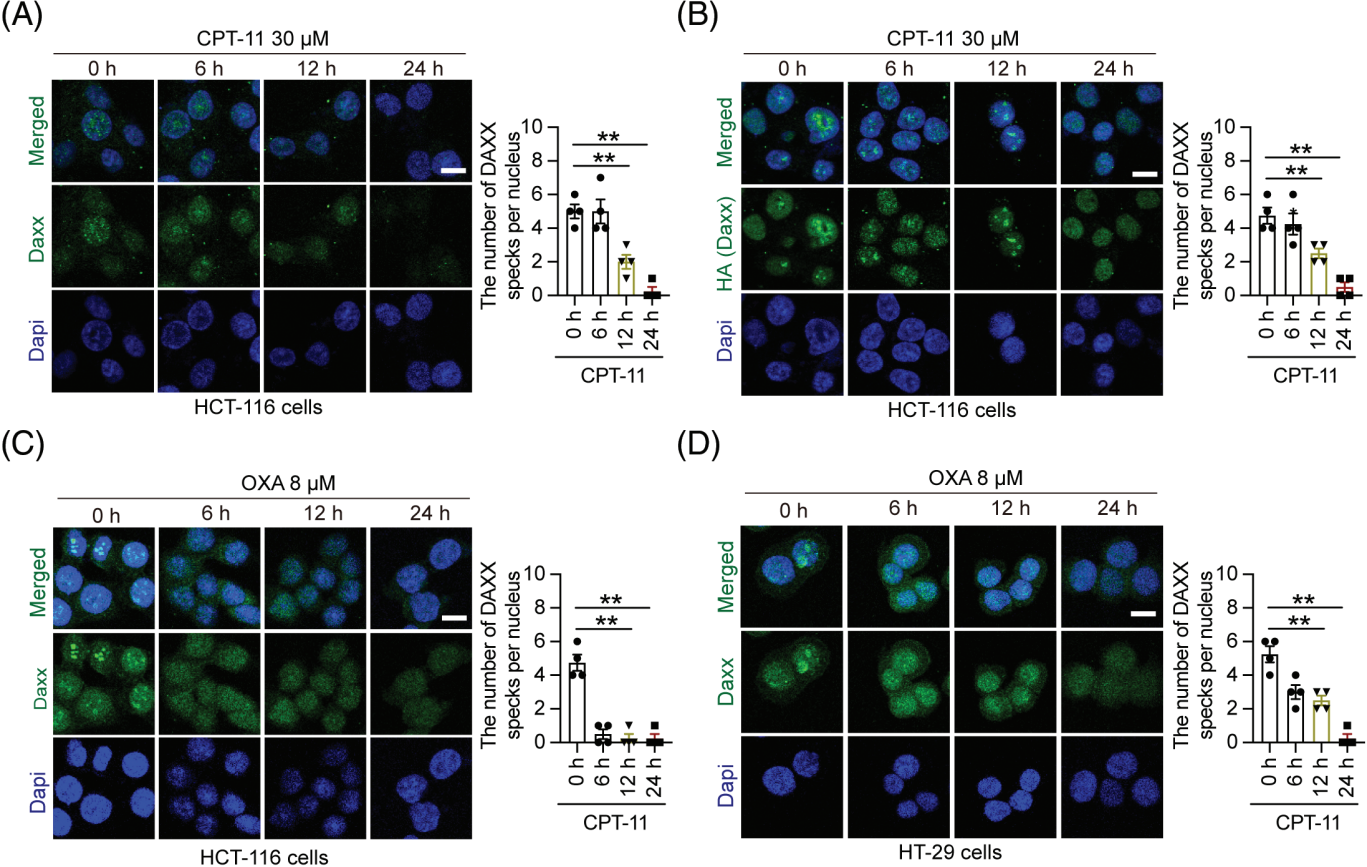
Chemotherapy inhibited the speck formation of Daxx in the nucleus. (A) HCT-116 cells were treated with CPT-11 (30 µM) for 0, 6, 12 and 24 h. (B) HCT-116 cells transfected with HA-Daxx plasmid were exposed to CPT-11 (30 µM) for 0, 6, 12, and 24 h. (C and D) HCT-116 and HT-29 cells were incubated with OXA (8 µM) for 0, 6, 12 and 24 h. The cellular immunofluorescence was used to analyze Daxx speck formation in the nucleus. For quantitative analysis of the Daxx speck, we randomly selected four fields to count the average number of Daxx specks in the nucleus. Scale bar: 50 µm. The circles, triangles, and squares represent different groups, and one group has only one shape. Values were shown as the means ± SEM in A–D. **p < 0.01 *vs*. as indicated.

Our previous results indicated that chemotherapeutic drugs promote Daxx degradation, however the underlying mechanisms of it remain unclear. Here, we used immunofluorescence to detect the location and expression of Daxx. In HCT-116 cells, our results revealed the presence of numerous specks in the nucleus, and that CPT-11 suppressed speck formation in a time-dependent manner (Fig. 2A). When we transfect exogenous Daxx into HCT-116 cells, the number of Daxx specks decreased after CPT-11 administration (Fig. 2B). Another chemotherapeutic drug, OXA, also inhibit speck formation in HCT-116 and HT-29 cells (Fig. 2C,D). Therefore, OXA and CPT-11 inhibited speck formation in human CRC cells.

### Daxx limited tumor cell migration and proliferation caused by chemotherapy

**Figure 3:**
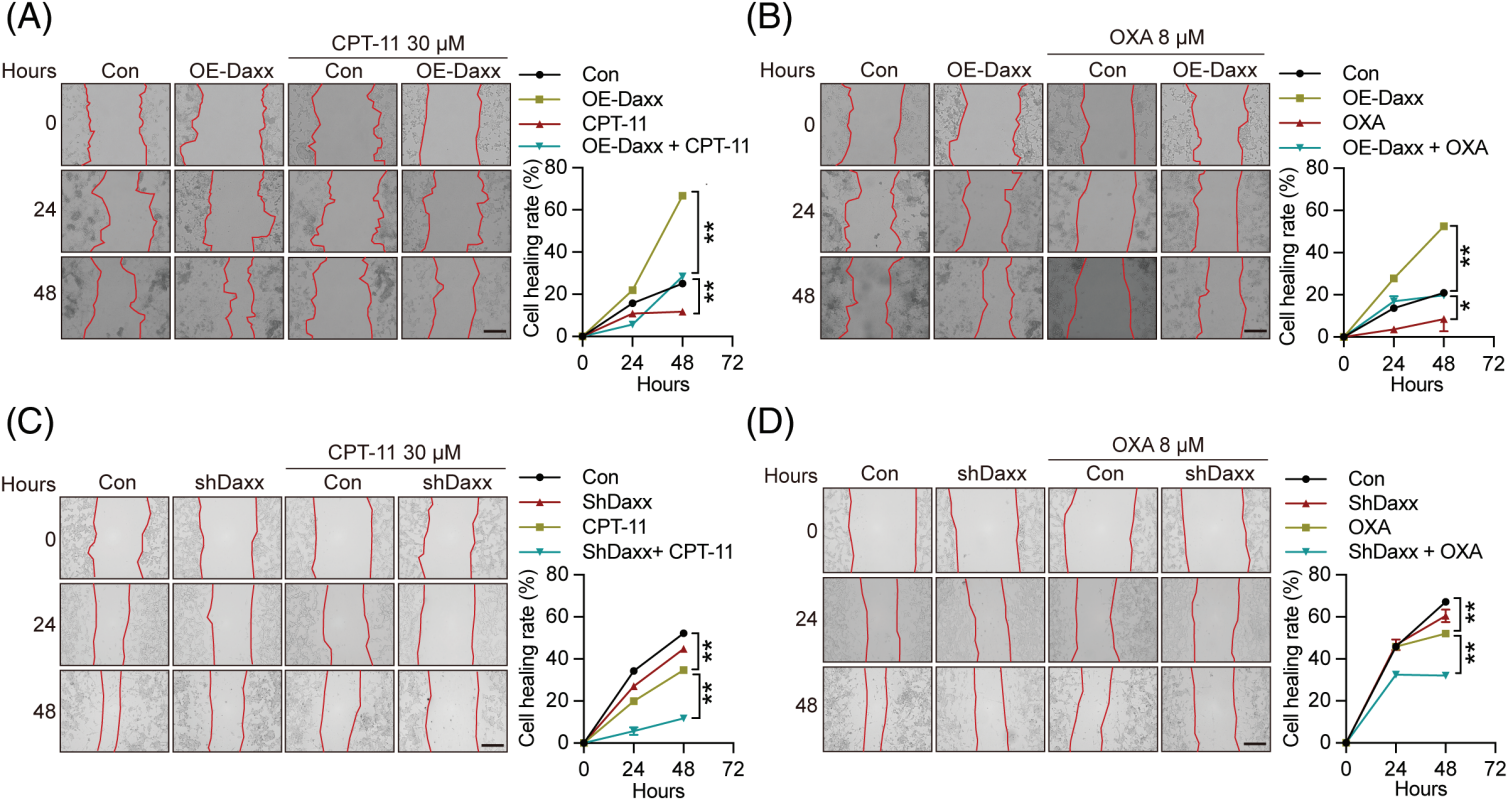
Daxx knock-down augmented chemotherapy-induced migration inhibition of colon cancer cells. (A and B) HCT-116 cells transfected with HA-Daxx by lip2000 were treated with CPT-11 (30 µM) or OXA (8 µM) for 24 and 48 h. Scale bar: 50 µm. (C and D) CT-26 cells infected with lentivirus-shDaxx were exposed to CPT-11 (30 µM) or OXA (8 µM) for 0, 24, and 48 h. The cellular picture was captured by microscope. Scale bar: 50 µm. Values were shown as the means ± SEM in A–D. *p < 0.05, **p < 0.01 *vs*. as indicated.

**Figure 4:**
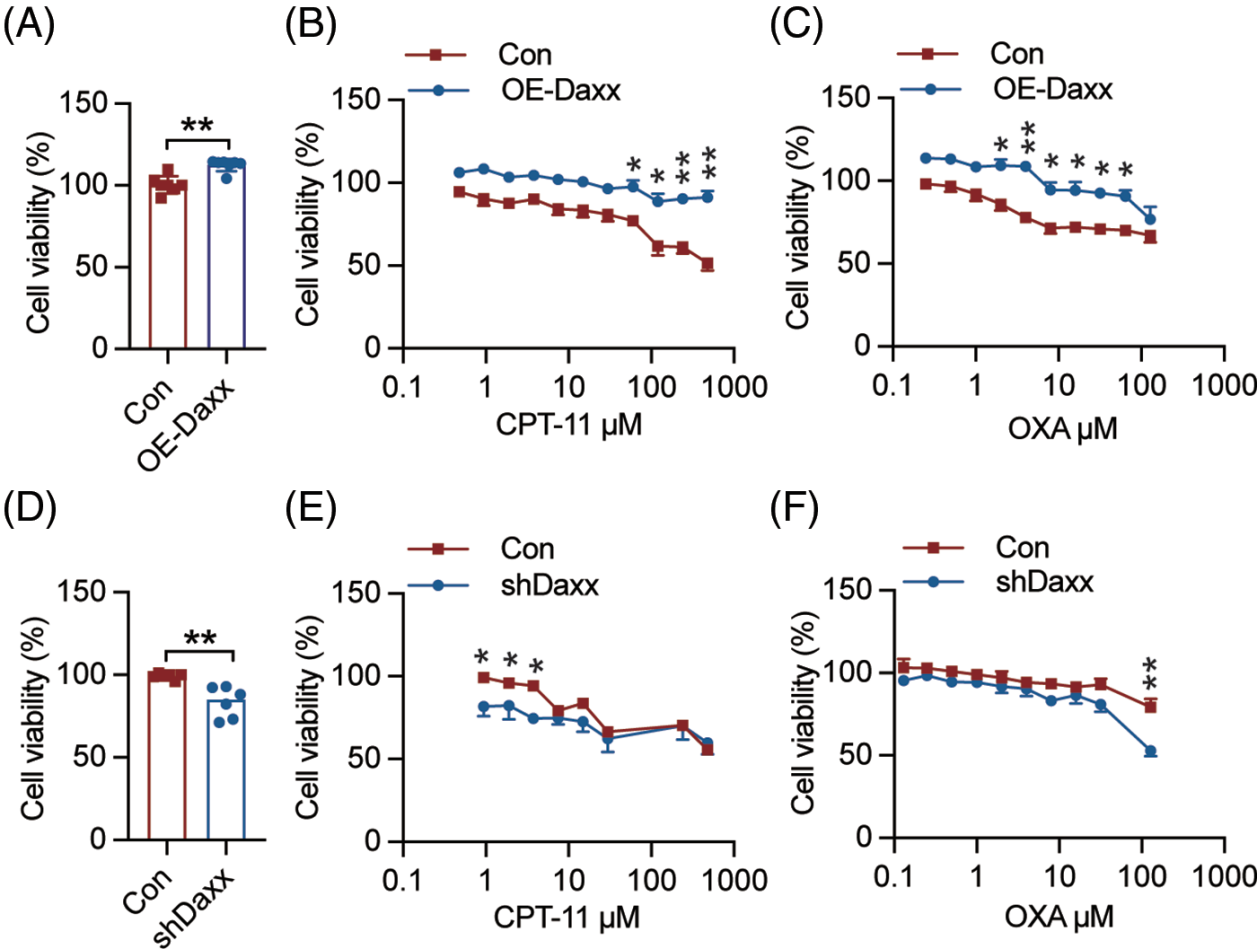
Daxx knock-down potentiated chemotherapy-induced proliferation inhibition of colon cancer cells. (A) The HA-Daxx plasmid was transfected to HCT-116 cells for 48 h, and an MTT assay was used to detect cell viability. (B and C) HCT-116 cells were transfected with HA-Daxx plasmid and treated with different concentrations of CPT-11 and OXA for 48 h. (D) HCT-116 cells were infected with lentivirus-shDaxx for 48 h. (E and F) HCT-116 cells were infected with lentivirus-shDaxx and treated with different concentrations of CPT-11 and OXA for 48 h. Values were shown as the means ± SEM in A–F. * *p* < 0.05, ** *p* < 0.01 *vs*. as indicated.

It is recognized that OXA and CPT-11 hinder tumor cellular migration and proliferation *in vivo* and *in vitro* [36,38]. We transfected Daxx over-expressing plasmids into HCT-116 cells to analyze Daxx’s effect on migration and proliferation. Here, overexpression of Daxx inhibited tumor cell migration induced by OXA and CPT-11 in HCT-116 cells (Fig. 3A,B). Similarly, Daxx knockdown in CT-26 cells markedly augmented the inhibitory migration caused by OXA and CPT-11 (Fig. 3C,D). In contrast, overexpression of Daxx restricted the suppression of tumor cell proliferation induced by OXA and CPT-11 in HCT-116 cells (Fig. 4A–C), while knockdown of Daxx enhanced OXA- and CPT-11-induced suppression of cellular proliferation in CT-26 cells (Fig. 4D–F). These results suggest that Daxx inhibited chemotherapy-induced migration and proliferation inhibition in human CRC cells.

### Daxx repressed cGAS-STING signal activation and promoted DNA damage repair

**Figure 5:**
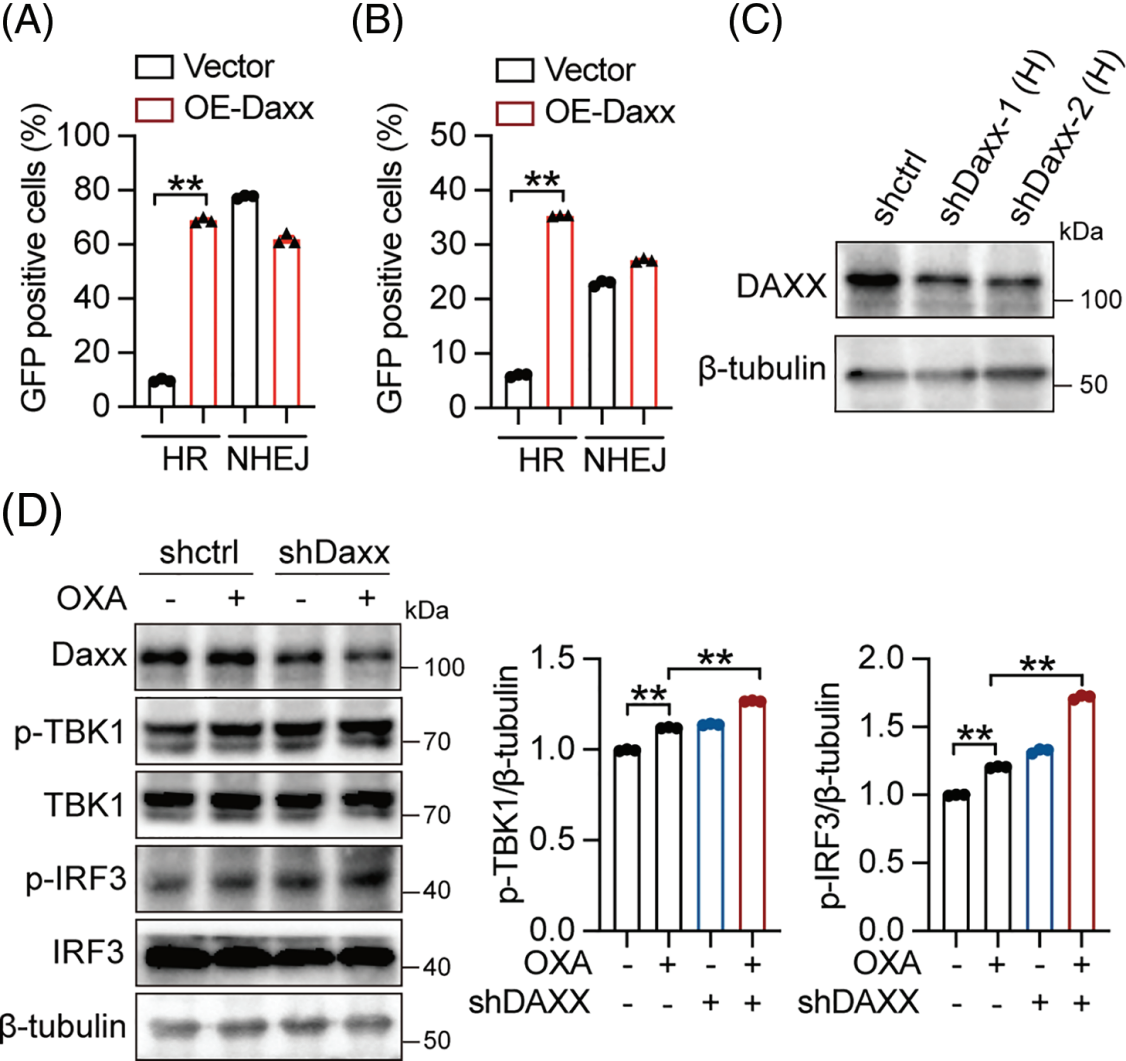
Daxx repressed cGAS-STING signal activation and promoted DNA damage repair. (A and B) HCT-116 and HEK293T cells were co-transfected with plasmids encoding pCBASceI, pDRGFP, pimEJ5GFP, and HA-Daxx, and flow cytometry was used to analyze the percent of GFP positive cells (Data were analyzed by FlowJo 10.0). (C) HCT-116 cells underwent a 48-h infection with lentivirus-shDaxx, followed by an analysis of Daxx expression levels using Western blotting. (D) HCT-116 cells were infected with lentivirus-shDaxx and treated with OXA (8 µM) for 12 h, the expression levels of p-TBK1, TBK1, p-IRF3, and IRF3 were detected by Western blotting. The circles, triangles, and squares represent different groups, and one group has only one shape. Densitometric analyses were shown. Values were shown as the means ± SEM in A–D. ** *p* < 0.01 *vs*. as indicated.

The cGAS-STING signal is crucial for antitumor immunity in tumor cells and immune-competent mice. Previous studies reported that chemotherapy drugs promote dsDNA production, which activates the DNA sensing pathway [39]. Homologous recombination (HR) and non-homologous end-joining (NHEJ) are two essential approaches to repairing genome double-strand breaks [40,41]. Our findings demonstrate that Daxx overexpression significantly increases the percent of GFP-positive cells in HCT-116 cells and HEK293T cells transfected with plasmids encoding pCBASceI and pDRGFP and fail to increase the percent of GFP-positive cells in HCT-116 cells and HEK293T cells transfected with plasmids encoding pCBASceI and pimEJ5GFP, suggesting that Daxx promotes HR and fail to affect NHEJ processes in HCT-116 and HEK293T cells (Fig. 5A,B). Additionally, Western blot results indicated that genetic ablation of Daxx promoted the phosphorylation of TBK1 and IRF3 proteins induced by OXA in HCT-116 cells (Fig. 5C,D), suggesting that Daxx limits STING pathway activation. In conclusion, Daxx promotes DNA damage repair via HR and limits cGAS-STING activation.

### Daxx knockdown promoted the antitumor effect of chemotherapy in a murine model of CRC

**Figure 6:**
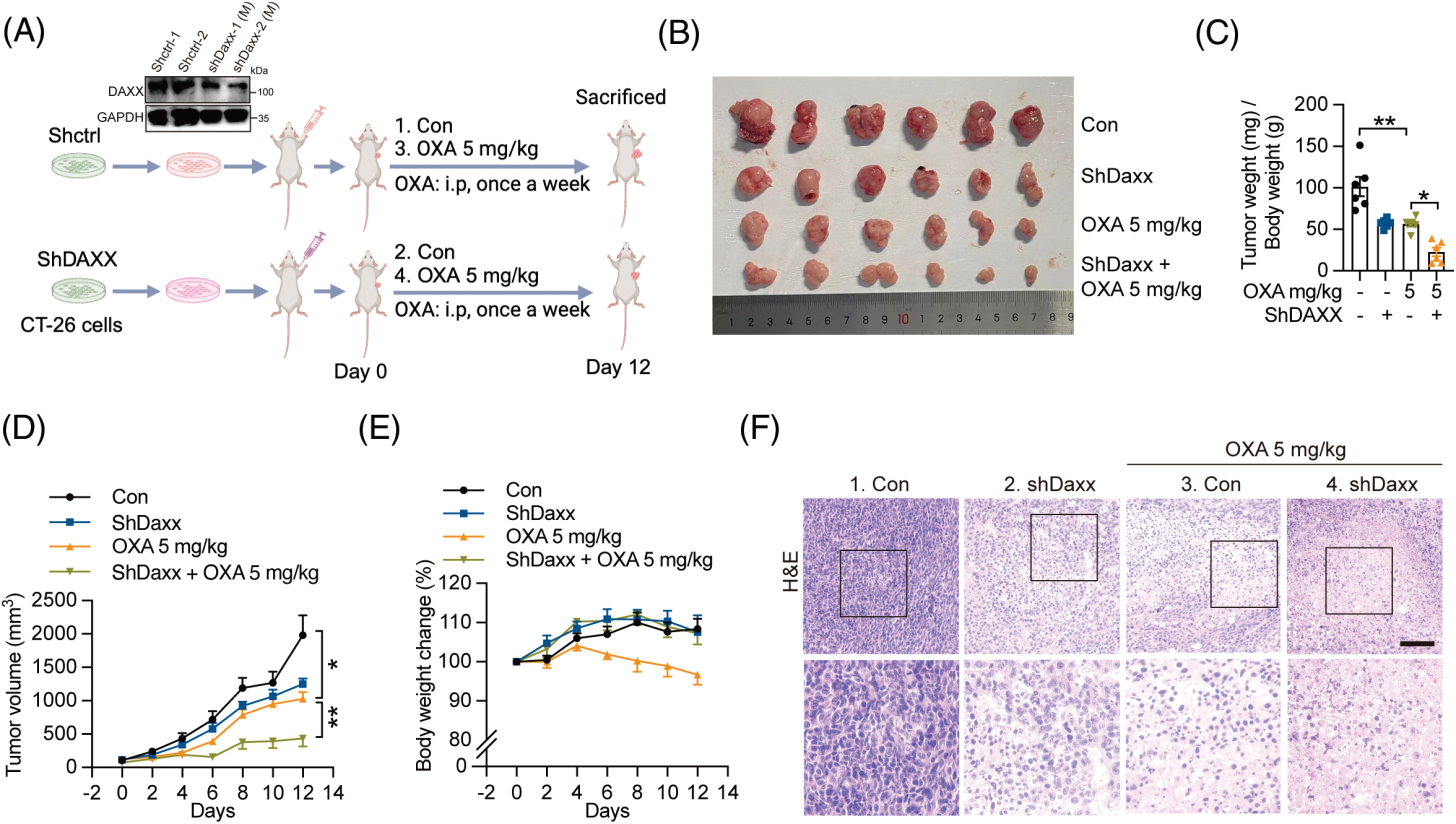
Daxx knock-down enhanced the antitumor effect of chemotherapy. (A) Schematic of the experimental procedure. The mice implanted with 1 × 10^6^ CT-26 cells infected lentivirus-shDaxx or shctrl (1 × 10^7^/mL in 1×PBS) were treated with OXA (5 mg/kg, i.p. once a week). (B and C) The picture of the tumor and the tumor weight. (D) Tumor volume. (E) The body weight of mice. (F) The H&E staining of tumor sections. n = 6 per group. Scale bar: 50 µm (top) and 20 µm (bottom). The circles, triangles, and squares represent different groups, and one group has only one shape. Values were shown as the means ± SEM in C, D. *p < 0.05, **p < 0.01 *vs*. as indicated.

To investigate whether Daxx mediates chemotherapy, we knocked down Daxx in CT-26 cells to evaluate its effects on tumor growth. Our results indicated that genetic ablation of Daxx in mouse tumor cells augmented OXA-induced tumor growth inhibition *in vivo* (Fig. 6A,B). Analysis of tumor weights and tumor size indicated that the tumor weights in the shDaxx combined with OXA groups were lower than those in the OXA group (Fig. 6C), with the tumor volumes following the same trends (Fig. 6D). Notably, genetic ablation of Daxx improved mouse body weight compared to the OXA group (Fig. 6E). H&E staining results showed clear nuclear shrinkage in the shDaxx combined with the OXA group compared with the OXA group (Fig. 6F). In summary, Daxx knockdown in tumor cells enhanced the antitumor ability of chemotherapy *in vivo*.

### Daxx knock-down augmented chemotherapy-induced cGAS-STING activation and immune responses in vivo

**Figure 7:**
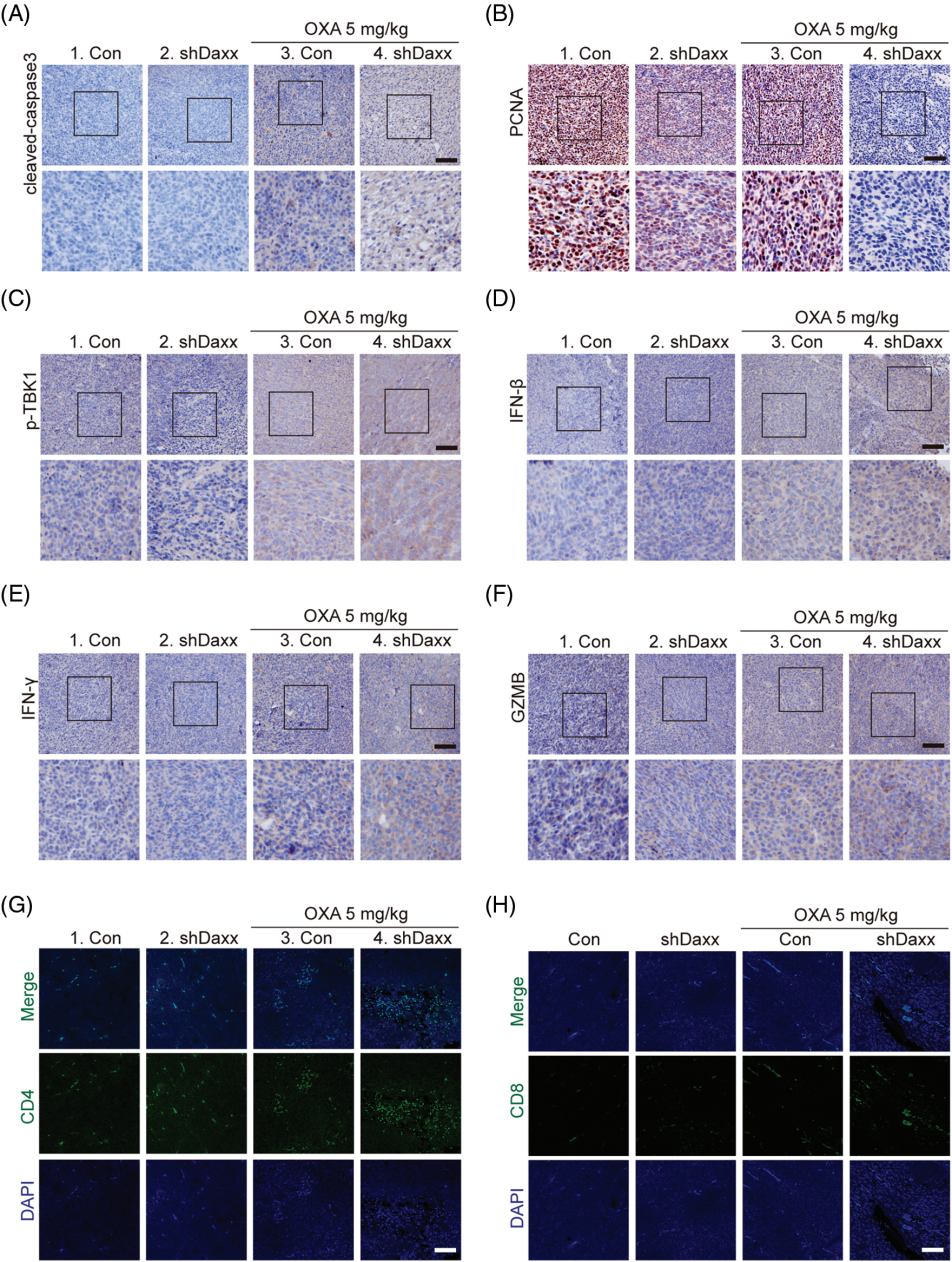
Daxx knock-down augmented chemotherapy-induced cGAS-STING activation and immune response *in vivo*. (A–F) The mice were implanted with 1 × 10^6^ CT-26 cells infected lentivirus-shDaxx or shctrl (1 × 10^7^/mL in 1×PBS), and the mice were treated with OXA (5 mg/kg, i.p. once a week) and finally sacrificed. Immunohistochemical analysis for cleaved-caspase3 (A), PCNA (B), p-TBK1 (C), IFN-β (D), IFN-γ (E), and GZMB (F). Scale bar: 50 µm (top) and 20 µm (bottom). (G and H) CD4 and CD8 T cells staining in tumor sections. Scale bar: 50 µm.

DNA sensor, the cGAS-STING signal, is an essential innate immune signaling pathway that senses dsDNA to mediate tumor progression and prognosis. Cleaved-caspase3 and PCNA are key makers of apoptosis and proliferation. Our results showed that higher expression levels of cleaved-caspase3 and PCNA were observed in the shDaxx combined with the OXA group than in the OXA group in the tumor sections, indicating that Daxx may promote tumor cell proliferation under OXA treatment (Fig. 7A,B). For the detection of cGAS-STING signal activation *in vivo*, the shDaxx combined with the OXA group shows elevated expressions of p-TBK1, IFN-β, IFN-γ, and GZMB compared with the single OXA group, suggesting that Daxx knock-down triggered STING-mediated interferon signals and immune responses to inhibit tumor growth (Fig. 7C–F). CD4+ and CD8+ T cells govern the host’s anti-tumor immune which is related to cGAG-STING signal activation. Our results indicated that the number of CD4+ and CD8+ T cells increased in the shDaxx combined with the OXA group compared with the OXA group (Fig. 7G,H), which suggests that knockdown of Daxx enhances the immune response by increasing the T cells’ number. In conclusion, our data indicate that knockdown Daxx in CRC cells augments chemotherapy by promoting STING-mediated interferon activation *in vivo*.

## Discussion

The cGAS-STING signal recognizes inherent and extrinsic nucleotides and has a crucial effect on viral infections, bacterial infections, organ fibrosis, autoimmune diseases, and cancer [35,39,42,43]. This signaling function is a double-edged sword, as STING-mediated interferon activation also leads to organ inflammation and fibrosis [44]. However, patients with tumors exhibit lower levels of STING signaling *in vivo* compared to healthy humans, suggesting that STING activation by agonists and endogenous molecules could improve the antitumor response [45]. Evidence has shown that STING is frequently functionally suppressed, and low STING expression predicts a bad prognosis in human cancers, including CRC [46,47]. Moreover, the cGAS-STING signal is essential to regulate the antitumor response of PD-L1 blockade, whereas intramuscular delivery of cGAMP constricts melanoma growth and prolonged survival [48,49]. In another study, a STING agonist decreased ascites accumulation and tumor size caused by a combination of carboplatin and anti-PD-1 antibodies in high-grade serous ovarian cancer [50,51]. Our results indicate that genetic ablation of Daxx up-regulated the p-TBK1 and p-IRF3 expression levels to sensitize chemotherapy, suggesting that Daxx is a negative regulator of the STING signal, thereby slowing tumor growth *in vivo*. However, the specific mechanism by which Daxx regulates cGAS-STING signaling remains unclear. One approach for STING activation involves the MRE11 (Double-strand break repair protein MRE11)–RAD50 (DNA repair protein RAD50)–NBN (Nibrin) complex liberating cGAS from nucleosome sequestration [52]. Since Daxx has many biological functions and exists in the nucleus, we hypothesized that Daxx may limit cGAS movement from the nucleus into the cytoplasm, thereby inhibiting STING activation.

Daxx is an essential protein that binds to Fas in the cytosolic domain and links its receptor to the apoptotic signal involved with JNK activation. Research has indicated that promyelocytic leukemia protein (PML) influences cellular proliferation and tumor inhibition through the modulation of apoptotic signals, and deactivation of PLM completely abrogates the proapoptotic ability of Daxx [53]. Our findings showed that Daxx knockdown inhibits the migration and proliferation of CRC cells, which is consistent with previous results. However, some studies have also demonstrated that Daxx mutations can increase apoptosis and either directly or indirectly suppress apoptosis in early embryos *in vivo* [54]. In our research, Daxx promoted colon cancer cell migration and proliferation, independently of the Fas-mediation cell death signal. Instead, its effect was associated with other death pathways, including Tumor necrosis factor α receptor (TNFαR), Death receptor 3 (DR3), Death receptor 4 (DR4), and Death receptor 5 (DR5).

Chemotherapy and radiotherapy are essential for slowing the progression of CRC [55]. Some studies have demonstrated resistance to chemotherapeutic drugs (CPT-11 and OXA) in CRC patients, yet the mechanisms of this resistance remain elusive. Our *in vitro* experimental results indicated that CPT-11 and OXA promote the degradation of the Daxx protein. Genetic ablation of Daxx sensitized chemotherapy, suggests that Daxx may be considered an essential marker for the prognosis and resistance of CRC patients. Additionally, a new approach that disrupts Daxx speck formation in the nucleus has been proposed to overcome chemotherapy resistance. Thus, we surmised that Daxx is a key indicator of the mechanism of chemotherapy resistance, potentially influencing the prognosis of patients with CRC.

However, our research has some limitations. The *in vivo* and *in vitro* detailed mechanisms of Daxx regulation of the cGAS-STING pathway and the DNA damage repair approach need to be explored. Daxx expression levels in tumor samples from patients with CRC who are resistant to chemotherapy compared with sensitive patients are not clear. whether Daxx protects nuclear membrane integrity to regulate DNA release into the cytoplasm and cGAS modification remains indeterminate.

## Conclusions

In conclusion, chemotherapeutic drugs (CPT-11 and OXA) promote the degradation of Daxx. Both inhibited the speck formation of Daxx, and genetic ablation of Daxx synergistically inhibited tumor growth caused by chemotherapy *in vivo*. Daxx promotes DNA damage repair through homologous recombination and inhibits cGAS-STING signal activation, suggesting that Daxx impairs colon cancer chemotherapy by restricting the cGAS-STING signal activation to mediate antitumor immune responses.

## Data Availability

The datasets analyzed during the current study are available from the corresponding author upon reasonable request.
